# Spondyloarthritis in First-Degree Relatives and Spouses of Patients with Inflammatory Bowel Disease: A Nationwide Population-based Cohort Study from Sweden

**DOI:** 10.1093/ecco-jcc/jjae041

**Published:** 2024-03-22

**Authors:** Sarita Shrestha, Judith S Brand, Mehdi Osooli, Carl Eriksson, Ida Schoultz, Johan Askling, Tine Jess, Scott Montgomery, Ola Olén, Jonas Halfvarson

**Affiliations:** School of Medical Sciences, Faculty of Medicine and Health, Örebro University, Örebro, Sweden; Population Health Sciences, Bristol Medical School, University of Bristol, Bristol, UK; Medical Research Council Integrative Epidemiology Unit, University of Bristol, Bristol, UK; Clinical Epidemiology and Biostatistics, School of Medical Sciences, Faculty of Medicine and Health, Örebro University Hospital, Örebro, Sweden; Clinical Epidemiology Division, Department of Medicine Solna, Karolinska Institutet, Stockholm, Sweden; Clinical Epidemiology Division, Department of Medicine Solna, Karolinska Institutet, Stockholm, Sweden; Department of Gastroenterology, Faculty of Medicine and Health, Örebro University, Örebro, Sweden; School of Medical Sciences, Faculty of Medicine and Health, Örebro University, Örebro, Sweden; Clinical Epidemiology Division, Department of Medicine Solna, Karolinska Institutet, Stockholm, Sweden; Rheumatology, Theme Inflammation and Ageing, Karolinska University Hospital, Stockholm, Sweden; Center for Molecular Prediction of Inflammatory Bowel Disease [PREDICT], Department of Clinical Medicine, Aalborg University, Copenhagen, Denmark; Department of Gastroenterology & Hepatology, Aalborg University Hospital, Aalborg, Denmark; Clinical Epidemiology and Biostatistics, School of Medical Sciences, Faculty of Medicine and Health, Örebro University Hospital, Örebro, Sweden; Clinical Epidemiology Division, Department of Medicine Solna, Karolinska Institutet, Stockholm, Sweden; Department of Epidemiology and Public Health, University College London, London, UK; Clinical Epidemiology Division, Department of Medicine Solna, Karolinska Institutet, Stockholm, Sweden; Department of Clinical Science and Education Södersjukhuset, Karolinska Institutet, Stockholm, Sweden; Sachs’ Children and Youth Hospital, Stockholm South General Hospital, Stockholm, Sweden; Department of Gastroenterology, Faculty of Medicine and Health, Örebro University, Örebro, Sweden

**Keywords:** Inflammatory bowel diseases, spondyloarthritis, first-degree relatives, spouses, epidemiology, ulcerative colitis

## Abstract

**Background and Aims:**

Register-based research suggests a shared pathophysiology between inflammatory bowel disease [IBD] and spondyloarthritis [SpA], but the role of familial [genetic and environmental] factors in this shared susceptibility is largely unknown. We aimed to compare the risk of SpA in first-degree relatives [FDRs] and spouses of IBD patients with FDRs and spouses of matched, population-based, reference individuals.

**Methods:**

We identified 147 080 FDRs and 25 945 spouses of patients with incident IBD [*N* = 39 203] during 2006–2016, and 1 453 429 FDRs and 258 098 spouses of matched reference individuals [*N* = 390 490], by linking nationwide Swedish registers and gastrointestinal biopsy data. Study participants were followed 1987–2017. Cox regression was used to estimate hazard ratios [HRs] of SpA.

**Results:**

During follow-up, 2430 FDRs of IBD patients [6.5/10 000 person-years] and 17 761 FDRs of reference individuals [4.8/10 000 person-years] were diagnosed with SpA, corresponding to an HR of 1.35 [95% CI:1.29, 1.41]. In subgroup analyses, the increased risk of SpA was most pronounced in FDRs of Crohn’s disease patients [HR = 1.44; 95% CI:1.34,1.5 6] and of IBD patients aged <18 years at diagnosis [HR = 1.46; 95% CI: 1.27, 1.68]. IBD patients’ spouses also had a higher SpA rate than reference individuals’ spouses, but the difference was less pronounced [4.3 vs 3.5/10 000 person-years; HR = 1.22; 95% CI:1.09, 1.37]. No subgroup-specific risk pattern was identified among spouses.

**Conclusions:**

The observed shared familial risks between IBD and SpA support shared genetic factors in their pathogenesis. However, spouses of IBD patients were also at increased risk for SpA, reflecting the influence of environmental exposures or similarities in health-seeking patterns.

## Graphical Abstract

**Graphical Abstract:**
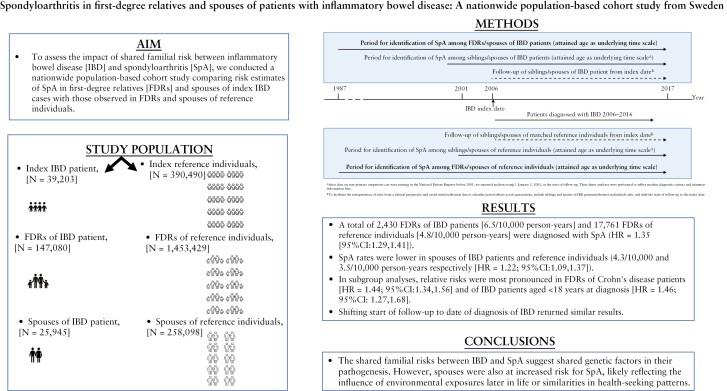

## 1. Introduction

Inflammatory bowel disease [IBD] is an idiopathic, chronic, inflammatory disorder of the gastrointestinal tract that comprises Crohn’s disease, ulcerative colitis, and IBD-unclassified [IBD-U].^1,2^ Extraintestinal manifestations, ie, inflammation affecting organ systems outside the gut, are common in IBD.^3^ One of the most common extraintestinal manifestations is spondyloarthritis [SpA], including ankylosing spondylitis, psoriatic arthritis, enteropathic arthritis, reactive arthritis, and undifferentiated SpA.^4–7^ Recent findings from a Swedish register-based study indicate that patients with IBD are more likely to develop SpA than matched reference individuals from the general population, both before and after the IBD diagnosis, supporting evidence of a shared pathophysiology.^8^

IBD and SpA are complex genetic disease entities, with estimated heritabilities of 67–75% for IBD and 72–82% for ankylosing spondylitis, one of the most well-studied forms of SpA.^9,10^ Additional support for a shared genetic basis between IBD and SpA comes from an Icelandic genealogy database study demonstrating familial clustering of both diseases, with a risk ratio for ankylosing spondylitis of 3.0 in first-degree relatives [FDRs] of patients with IBD.^11^ Immunochip studies have identified 20 shared genetic loci between ankylosing spondylitis and IBD to date, and at almost all of these loci, the lead single nucleotide polymorphism [SNP] correlated between ankylosing spondylitis and IBD.^12^ Besides a common genetic background, both diseases may also have environmental risk factors in common. A decreased bacterial diversity, and an altered gut microbiota composition that resembles IBD-associated dysbiosis, have been observed in patients with SpA.^13–16^ Similarly, a potential role of vitamin D as a regulator of the innate and adaptive immune response has been highlighted in both IBD^17,18^ and SpA,^19^ even though evidence for a shared causal link is sparse.

If the excess risk of SpA in patients with IBD was explained by shared susceptibility, one would expect an increased risk of SpA also in individuals without IBD but with a similar exposure to genetic and/or environmental risk factors, such as their FDRs. In contrast to FDRs, spouses do not share the same genetic factors and only share environmental factors later in life, and if the excess risk of SpA was explained by exposure to environmental risk factors at adult age, one would expect an increased risk of SpA also in spouses of patients with IBD. Few studies have examined familial risks at the population level or whether such risks vary according to subtype of IBD [Crohn’s disease and ulcerative colitis] or age at IBD diagnosis.^11^ To assess the shared familial risk between IBD and SpA and to disentangle the potential influence of genetic and environmental risk factors, we conducted a nationwide, population-based cohort study comparing the absolute and relative risk estimates of SpA among FDRs and spouses of index IBD patients, with those observed in FDRs and spouses of reference individuals from the general population.

## 2. Methods

### 2.1. Setting and data sources

Sweden is a high-income country with a population of 10 million inhabitants. The health care system in Sweden is tax-funded and access to health care is universal.^20^ Patients with IBD are usually diagnosed and treated by gastroenterologists or internists, and patients with SpA are seen by rheumatologists or internists for diagnosis and for anti-rheumatic treatment, typically in hospital-based, outpatient care settings. All residents of Sweden are assigned a personal identity number at birth or immigration which allows linkage between nationwide registers with data on demographics, FDRs [parents, siblings, and offspring], spouses, morbidity, and mortality with virtually no loss to follow-up. Detailed information on the registers used as the source of data in this study are included in the Supplementary material, including Supplementary Figure 1.

### 2.2. Study population

#### 2.2.1. Identification of IBD patients and general population controls

We used relevant International Classification of Diseases [ICD] codes [Supplementary Table 1] to identify patients with a diagnosis of IBD in the Swedish National Patient Register [NPR] [containing records of inpatient care since 1964 and non-primary outpatient care since 2001]. Patients diagnosed with incident IBD [*N* = 39 203] between January 1, 2006, and December 31, 2016, were identified and followed until 31 December, 2017.^8^ Patients with IBD had to have at least two records of IBD in the NPRs, or one such record plus a colorectal biopsy report with a morphology code suggestive of IBD, from the Swedish Epidemiology strengthened by histopathology reports in Sweden [ESPRESSO] Biopsy Register.^21^ For each incident IBD diagnosis [ie, individuals who were diagnosed with IBD during the study period] we identified up to 10 reference individuals [*N *= 390 490] from the general population matched by birth year, sex, area of residence, and calendar year in the national population register in Sweden.^22^ Reference individuals had to be alive and free of IBD at the index date [ie, the date of the first IBD-associated ICD diagnostic code from the NPR and Systematized Nomenclature of Medicine [SNOMED] codes from the ESPRESSO database in the index IBD patient].

#### 2.2.2. First-degree relatives and spouses of IBD patients and general population controls

We identified all biological FDRs [parents, siblings, and offspring] of IBD patients and reference individuals through the Swedish Multi-generation Register. The Swedish Multi-generation Register includes information on people born from 1932 onwards and any relationship to biological and adoptive parents and those who are registered as residents in Sweden after 1961.^23^ Registered spouses [defined through marriage; a person who at some point was married to an IBD patient or reference individual] were identified by linking data from the Swedish Total Population Register.

### 2.3. Spondyloarthritis

We retrieved diagnoses of SpA including ankylosing spondylitis, psoriatic arthritis, enteropathic arthritis, reactive arthritis, and undifferentiated spondyloarthritis from the NPR. For this study, we extracted the first in- or outpatient diagnosis encountered based on relevant ICD-codes [ie, 9th and 10th revision, applying to diagnoses made 1987–1996 and 1997 onwards, respectively] for the historically categorised, phenotypic subtypes of SpA [Supplementary Table 2]. Consistent with previous validation studies,^24–26^ a single occurrence of a SpA diagnosis was deemed sufficient to qualify for an event in the analyses.

### 2.4. Statistical analysis

The incidence rate of SpA in FDRs and spouses of index IBD patients was compared with the incidence rate in FDRs and spouses of the reference individuals [ie, exposure is defined as being an FDR or spouse of a patient with IBD]. Next, we used Cox proportional hazards models to estimate associations between the exposure [being an FDR of an IBD index patient] and outcome [diagnosis of SpA], using attained age as underlying time scale [Supplementary Figure 2]. Follow-up started from January 1, 1987 [the start year of the Swedish Patient Register], or the date of birth for those born after this date and ended at first diagnosis of SpA, death, emigration, or December 31, 2017, whichever occurred first. If a reference individual from the general population was diagnosed with IBD during follow-up, the FDRs and possible spouses of the reference individual were censored at the date of IBD diagnosis. All hazard ratios [HRs] with 95% confidence intervals [CIs] were adjusted for sex and calendar year of birth. *P-*values for interaction terms were derived from Cox regression, including all matching factors as covariates in the models. Analyses were performed for IBD combined, including all patients with Crohn’s disease, ulcerative colitis, and IBD-U, and for Crohn’s disease and ulcerative colitis separately.

In FDRs, we further examined HRs by type of relationship [parent, sibling, and offspring], under the assumption that spouses are more likely to be exposed to the same environmental factors and for a longer duration of time with advancing age. Possible variation in HRs by attained age was therefore evaluated through tests of proportional hazards using scaled Schoenfeld residuals.

### 2.5. Sensitivity analyses

We conducted a series of sensitivity analyses. First, since data on non-primary outpatient care were missing in the NPR before 2001, we repeated analyses of siblings and spouses using January 1, 2001, as the start of follow-up. These latter analyses were performed to minimise misclassification due to calendar period effects across generations and information bias, and to reflect modern diagnostic criteria. Second, in order to facilitate the interpretation of risks from a clinical perspective, we also reiterated this sensitivity analysis shifting start of follow-up from January 1, 2001, or later, to date of entry of the index individual [ie, the index date, as the index date refers to the time point when it was first known whether a proband had IBD. In this later analysis, HRs were adjusted for sex, age, and calendar year at the index date. Third, we repeated analyses excluding FDRs/spouses diagnosed with IBD during follow-up, to assess associations independently of a family history of IBD. Fourth, to examine the potential role of smoking in shared environmental risks between IBD and SpA, we restricted analyses to individuals without a chronic obstructive pulmonary disease [ie, a proxy for smoking]. Last, since prior evidence concerning shared familial risks mostly comes from studies examining IBD and ankylosing spondylitis, we also present results for this specific SpA diagnosis to facilitate comparison with the existing literature.

All analyses were performed in Stata 16.1 version.^27^

### 2.6. Ethical considerations

The study was approved by the Regional Ethics Committee in Stockholm in 2015 [approval numbers: 2007/785-31/5; 2011/1509-32; 2012/601-32; 2014/1287-31/4; 2015/0004-31; 2015/615-32; 2015/1030-32; 2016/191-31/2; 2023-04867-02-446856]. Informed consent was waived due to the strict registry-based nature of the study.^28^

## 3. Results

### 3.1. Characteristics of FDRs and spouses

We identified a cohort of 147 080 FDRs and 25 945 spouses of 39 203 IBD patients, and 1 453 429 FDRs and 258 098 spouses of 390 490 reference individuals [Table 1]. FDRs/spouses of IBD patients and reference individuals did not notably differ in terms of age, sex, and median follow-up time. As anticipated, FDRs of IBD patients [3.9%] more frequently had a diagnosis of IBD themselves than FDRs of reference individuals [1.3%].

**Table 1:** Characteristics of FDRs and spouses of patients with inflammatory bowel disease and of reference individuals from the general population, matched on sex, age, and place of residence.

	IBD/reference individuals	FDRs	Parents	Siblings	Children	Spouses
IBD patients						
*N*	39 203	147 080	44 770	50 247	52 063	25 945
Sex, % [*N*]						
Male	50.7 [19 863]	50.1 [73 685]	48.1 [21 517]	50.9 [25 598]	51.0 [26 570]	52.0 [13 364]
Female	49.3 [19 340]	49.9 [73 395]	51.9 [23 253]	49.1 [24 649]	49.0 [25 493]	48.0 [12 581]
Age at index date^a^						
Median [IQR]	37.0 [24.0-58.0]	43.6 [24.3-57.5]	56.7 [49.3-65.4]	37.6 [23.7-57.4]	25.0 [11.0-39.9]	47.7 [34.6-62.3]
Min-Max	0.0-100.0	[0.0-85.8]	[21.1-85.8]	[0.0-85.5]	[0.0-80.2]	[11.9-85.4]
Calendar year at index date						
Median [Min-Max]	2012 [2007-2017]	2012 [2007-2017]	2012 [2007-2017]	2012 [2007-2017]	2011 [2007-2017]	2012 [2007-2017]
IBD diagnosis, % [*N*]	-	3.9 [5689]	5.0 [2257]	4.5 [2283]	2.2 [1149]	1.8 [465]
Reference individuals						
*N*	390 490	1 453 429	437 629	491 156	524 644	258 098
Sex, % [*N*]						
Male	50.7 [197 878]	50.4 [732 518]	48.0 [210 104]	51.4 [252 485]	51.4 [269 929]	51.0 [131 708]
Female	49.3 [192 612]	49.6 [720 911]	52.0 [227 525]	48.6 [238 671]	48.5 [254 715]	49.0 [126 390]
Age at index date^a^						
Median [IQR]	37.0 [24.0-58.0]	43.2 [23.8-57.4]	56.9 [49.2-65.7]	37.8 [23.5-57.3]	24.4 [10.8-39.2]	47.8 [34.8-62.2]
Min-Max	0.0-100.0	[0.0-85.8]	[17.7-85.8]	[0.0-85.8]	[0.0-80.4]	[8.5-85.7]
Calendar year at index date						
Median [Min-Max]	2012 [2007-2017]	2012 [2007-2017]	2012 [2007-2017]	2012 [2007-2017]	2011 [2007-2017]	2012 [2007-2017]
IBD diagnosis, % [*N*]	-	1.3 [19 123]	1.6 [7199]	1.4 [7062]	0.9 [4862]	1.6 [4059]

IBD, inflammatory bowel disease; FDRs, first-degree relatives; *N*, number; IQR, interquartile range; Min, minimum; Max, maximum.

^a^For individuals who were born before the index date of IBD patients/reference individuals.

### 3.2. Risk of SpA in FDRs and spouses of IBD patients and reference individuals

A total of 2430 FDRs of IBD patients [6.5/10 000 person-years] and 17 761 [4.8/10 000 person-years] FDRs of reference individuals were diagnosed with SpA. Overall, the adjusted HR for SpA comparing FDRs of IBD patients to FDRs of reference individuals was 1.35 [95% CI: 1.29, 1.41] [Table 2].

**Table 2:** Risk estimates of SpA comparing FDRs/spouses of IBD patients with FDRs/spouses of reference individuals from the general population, overall and by subtype.

Exposure	FDRs of IBD patients [*N* with SpA/*N* total]	FDRs of Reference individuals [*N* with SpA/*N* total]	HR [95% CI]	Spouses of IBD patients [*N* with SpA/*N* total]	Spouses of Reference individuals [*N* with SpA/*N* total]	HR [95% CI]
** IBD**	2430/147 047	17 761/1 452 882	1.35 [1.29, 1.41]	327/25 945	2690/258 098	1.22 [1.09, 1.37]
** Crohn’s disease**	737/42354	5105/423 863	1.44 [1.34, 1.56]	84/7125	728/70 832	1.15 [0.92, 1.45]
** Ulcerative colitis**	1241/77 194	9228/760 723	1.32 [1.24, 1.40]	173/14 019	1438/139 592	1.21 [1.04, 1.42]

Hazard ratios were obtained from Cox proportional hazards models with attained age as underlying time scale, adjusting for sex and 10-year calendar period at birth date.

SpA, spondyloarthritis; CI, confidence interval; FDRs, first-degree relatives; HR, hazard ratio; *N*, number; IBD, inflammatory bowel disease.

As expected, rates of SpA were lower for spouses than for FDRs, but increased among spouses of IBD patients compared with spouses of reference individuals. In total, 327 SpA events [4.3/10 000 person-years] were observed in spouses of IBD patients, and 2690 SpA events [3.5/10 000 person-years] in spouses of reference individuals, resulting in an adjusted HR of 1.22 [95% CI: 1.09, 1.37] [Table 2]. There was no evidence of non-proportional hazards in spouses, meaning that the magnitude of associations did not vary by attained age. No IBD subtype-specific pattern was found for spouses [*P* for interaction between Crohn’s disease and ulcerative colitis = 0.75], but HRs for FDRs of patients with Crohn’s disease 1.44 [95% CI: 1.34, 1.56] seemed to be of somewhat higher magnitude than for FDRs of patients with ulcerative colitis (HR = 1.32 [95% CI: 1.24, 1.40]), [*P* for interaction = 0.07].

Table 3 shows the results by FDR relation type. Parents, siblings, and children of IBD patients were all at increased risk of SpA compared with FDRs of population reference individuals, with risk estimates being of similar magnitude [*P* for interaction = 0.68]. Neither did separate analyses for Crohn’s disease and ulcerative colitis reveal different magnitudes of HRs across generations [*P* for interaction = 0.15 and 0.51, respectively].

**Table 3:** Risk estimates of SpA comparing FDRs of IBD patients with FDRs of reference individuals from the general population by relation type.

Exposure	Relation	FDRs of IBD patients [*N* with SpA/*N* total]	FDRs of reference individuals [*N* with SpA/*N* total]	HR [95% CI]
**IBD**	**Parent** **^1^**	1056/44 770	7702/437 627	1.35 [1.27, 1.44]
	Mother	553/23 253	4150/227 524	1.31 [1.20, 1.43]
	Father	503/21 517	3552/210 103	1.40 [1.27, 1.53]
	**Sibling** **^1^**	842/50 231	6062/490 986	1.37 [1.27, 1.47]
	Sister	442/24 640	3082/238 595	1.40 [1.27, 1.54]
	Brother	400/25 591	2980/252 391	1.33 [1.20, 1.48]
	**Children** **^1^**	532/52 046	3997/524 269	1.31 [1.20, 1.44]
	Daughter	282/25 487	2072/254 536	1.32 [1.17, 1.50]
	Son	250/26 559	1925/269 733	1.30 [1.14, 1.49]
**Crohn’s disease**	**Parent** **^2^**	308/13 714	2324/135 852	1.33 [1.18, 1.50]
	Mother	167/7117	1258/70 509	1.33 [1.13, 1.56]
	Father	141/6597	1066/65 343	1.33 [1.11, 1.58]
	**Sibling** **^2^**	272/14 719	1725/145 822	1.59 [1.40, 1.81]
	Sister	148/7243	886/70 767	1.66 [1.40, 1.98]
	Brother	124/7476	839/75 055	1.51 [1.25, 1.82]
	**Children** **^2^**	157/13 921	1 056/142 189	1.46 [1.24, 1.73]
	Daughter	81/6859	534/69 075	1.47 [1.16, 1.85]
	Son	76/7062	522/73 114	1.45 [1.14, 1.85]
**Ulcerative colitis**	**Parent** **^3^**	552/22 884	3977/222 803	1.36 [1.24, 1.48]
	Mother	280/11 885	2161/116 027	1.27 [1.12, 1.44]
	Father	272/10 999	1816/106 776	1.46 [1.29, 1.66]
	**Sibling** **^3^**	424/26 030	3149/253 763	1.32 [1.19, 1.46]
	Sister	220/12 789	1608/123 216	1.32 [1.15, 1.52]
	Brother	204/13 241	1541/130 547	1.31 [1.13, 1.52]
	**Children** **^3^**	265/28 280	2102/284 157	1.25 [1.10, 1.42]
	Daughter	143/13 838	1109/138 012	1.26 [1.06, 1.50]
	Son	122/14 442	993/146 145	1.23 [1.02, 1.49]

Hazard ratios of SpA comparing FDRs of IBD patients to FDRs of reference individuals were obtained from Cox proportional hazards models with attained age as underlying time scale, adjusting for sex and 10-year calendar period at birth date.

SpA, spondyloarthritis; FDR, first-degree relative; HR, hazard ratio; CI, confidence interval; *N*, number; IBD, inflammatory bowel disease.

*P* for interaction by relation of FDRs [parents; sibling; children] in IBD^1^=0.79, CD^2^=0.15, UC^3^=0.51.

Results of age- and sex-stratified analyses of FDRs and spouses are presented in Table 4. HRs for FDRs of IBD patients decreased in magnitude with increasing age at diagnosis of the index IBD patient (HR = 1.46 [95% CI: 1.27, 1.68] for FDRs of IBD patients aged <18 years, HR = 1.39 [95% CI: 1.32, 1.46] for FDRs of IBD patients aged 18 to 60 years, and HR = 1.20 [95% CI: 1.09, 1.31]) for FDRs of IBD patients above 60 years of age [*P* interaction = 0.006]. By contrast, HRs for spouses of IBD patients did not notably vary by the age of the index IBD patient. Overall, results for FDRs and spouses did not differ by sex of the IBD index case.

**Table 4:** Risk estimates of SpA comparing FDRs/spouses of IBD patients with FDRs/spouses of reference individuals from the general population, stratified by age and sex of IBD index patient/reference individual.

Exposure		FDRs of IBD patients [*N* with SpA/*N* total]	FDRs of Reference individuals [*N* with SpA/*N* total]	HR [95% CI]	Spouses of IBD patients [*N* with SpA/*N* total]	Spouses of Reference individuals [*N* with SpA/*N* total]	HR [95% CI]
IBD	**Age group**						
	<18 years	223/13 936	1509/139 404	1.46 [1.27 1.68]	NA	NA	NA
	18 to 60 years	1669/105 821	11 858/1 044 751	1.39 [1.32 1.46]	226/18 622	1830/185 857	1.25 [1.09, 1.44]
	>60 years	538/27 290	4394/268 727	1.20 [1.09 1.31]	101/ 7200	856/70 980	1.16 [0.95, 1.43]
	*P* interaction			0.006			0.26
	**Sex**						
	Female	1270/74 246	9053/729 356	1.36 [1.29 1.45]	165/13 375	1301/131 910	1.27 [1.08, 1.49]
	Male	1160/72 801	8708/723 526	1.33 [1.25 1.41]	162/12 570	1389/126 188	1.18 [1.00, 1.39]
	*P* interaction			0.55			0.56
Crohn’s disease	**Age group**						
	<18 years	80/5642	613/56 251	1.29 [1.02 1.63]	NA	NA	NA
	18 to 60 years	503/29 701	3318/298 289	1.52 [1.39 1.67]	58/5158	495/51 864	1.20 [0.91, 1.57]
	>60 years	154/7011	1174/69 323	1.29 [1.09 1.53]	26/1917	231/18 440	1.09 [0.72, 1.63]
	*P* interaction			0.13			0.45
	**Sex**						
	Female	414/22 060	2658/219 480	1.53 [1.38 1.70]	44/3906	365/38 198	1.18 [0.86, 1.61]
	Male	323/20 294	2447/204 383	1.34 [1.20 1.51]	40/3219	363/32 634	1.13 [0.82, 1.57]
	*P* interaction			0.11			0.90
Ulcerative colitis	**Age group**						
	<18 years	89/5311	591/53 394	1.47 [1.17 1.83]	NA	NA	NA
	18 to 60 years	877/57 515	6397/565 840	1.35 [1.26 1.45]	120/10 243	1006/101 763	1.21 [1.00, 1.46]
	>60 years	275/14 368	2240/141 489	1.19 [1.05 1.35]	53/3730	430/37 327	1.23 [0.93, 1.64]
	*P* interaction			0.14			0.49
	**Sex**						
	Female	626/38 603	4712/378 985	1.29 [1.19 1.40]	87/7118	671/70 201	1.30 [1.04, 1.63]
	Male	615/38 591	4516/381 738	1.35 [1.24 1.47]	86/6901	767/69 391	1.13 [0.91, 1.42]
	*P* interaction			0.48			0.37

Hazard ratios of SpA comparing FDRs/spouses of IBD patients with FDRs/spouses of reference individuals were obtained from Cox proportional hazards models with attained age as underlying time scale, adjusting for sex and 10-year calendar period at birth date.

SpA, spondyloarthritis; FDRs, first-degree relatives; HR, hazard ratio; CI, confidence interval; *N*, number; NA, not available; IBD, inflammatory bowel disease.

### 3.3. Sensitivity analyses

HRs for siblings and spouses of IBD patients remained materially unchanged when reiterating analyses with January 1, 2001, as follow-up [Supplementary Tables 3 and 4]. Although the SpA rates observed were higher than those observed in analysis with follow-up starting from 1987, FDRs of IBD patients and reference individuals exhibited rates per 10 000 person-years of 9.8 and 7.1, respectively. The corresponding rates in spouses were 6.8 and 5.7, respectively.

Starting follow-up from diagnosis of the IBD patient and the corresponding date of the reference individual returned similar results. Similar to analyses with attained age as underlying time scale, the risk of SpA in siblings of IBD patients was increased (HR = 1.40 [95% CI: 1.23, 1.59]) as was the risk in spouses of IBD patients (HR = 1.19 [95% CI: 0.97, 1.47]) [Table 5 and Supplementary Table 5].

**Table 5:** Risk of SpA from the index date comparing siblings/spouses of IBD patients with siblings/spouses of reference individuals from the general population.

Exposure	Siblings of IBD patients [*N* with SpA/*N* total]	Siblings of reference individuals [*N* with SpA/*N* total]	HR [95% CI]	Spouses of IBD patients [*N* with SpA/*N* total]	Spouses of reference individuals [*N* with SpA/*N* total]	HR [95% CI]
**IBD**	268/45 663	1892/450 823	1.40 [1.23, 1.59]	98/24 195	827/242 936	1.19 [0.97, 1.47]
**Crohn’s disease**	86/13 433	550/134 415	1.57 [1.25, 1.97]	26/6647	206/66 552	1.26 [0.84, 1.90]
**Ulcerative colitis**	140/23 630	977/232 576	1.41 [1.18, 1.68]	58/13 092	463/131 644	1.26 [0.96, 1.66]

Hazard ratios of SpA comparing siblings/spouses of IBD patients with siblings/spouses of reference individuals were obtained from Cox proportional hazards models with time from the index date as underlying time scale, adjusting for age, sex, and calendar year at index date [years].

SpA, spondyloarthritis; HR, hazard ratio; CI, confidence interval; *N*, number; IBD, inflammatory bowel disease.

Patterns of associations were also not materially different after excluding FDRs and spouses with a diagnosis of IBD from the analyses [Supplementary Table 6]. Supplementary Table 7 shows results restricted to individuals without a chronic obstructive pulmonary disease [COPD] diagnosis. Overall, HRs were not materially different in this analysis compared with the main analysis. Separate analyses for ankylosing spondylitis only are presented in Supplementary Tables 8 and 9. HRs for ankylosing spondylitis in FDRs and spouses of IBD patients were of somewhat higher than or similar magnitude to the corresponding risks for SpA overall (HR for ankylosing spondylitis in FDRs of IBD patients was 1.57 [95% CI: 1.44, 1.71], and 1.36 [95% CI: 1.08, 1.70] in spouses).

## 4. Discussion

### 4.1. Main findings

In this Swedish, population-based, cohort study, we demonstrated that FDRs of patients with IBD are at increased risk of being diagnosed with SpA compared with FDRs of reference individuals from the general population. The increased risk was not conferred by FDRs having an increased risk of IBD themselves, and we further found evidence of the risk for SpA being more pronounced for Crohn’s disease than for ulcerative colitis. The finding that HRs of SpA were similar within and between generations point to genetic predisposition as the predominant explanatory factor for shared susceptibility. However, environmental exposures and/or health care-seeking behaviour also seemed to contribute to shared disease pathogenesis, since SpA was more frequently diagnosed in spouses of IBD patients compared with their corresponding reference group.

### 4.2. Comparison with previous literature

Few studies to date have examined the shared familial risks between IBD and SpA, and to our knowledge, existing data are limited to ankylosing spondylitis only.^11^ Thjodleifsson *et al*. reported a risk ratio of 3.0 [95% CI: 1.8, 4.6] for ankylosing spondylitis in FDRs of Icelandic patients with IBD, whereas no increased risk was observed in spouses of IBD patients [0.9; 95% CI: 0.0, 3.9].^11^ We studied the risk of ankylosing spondylitis in a large cohort of 147 080 FDRs and 25 945 spouses of IBD patients, and adjusted our analyses for age, sex, and calendar year of birth. By contrast, the authors of the Icelandic study included relatives of 1352 IBD patients and 205 ankylosing spondylitis patients only.^11^ The stronger familial risks observed in FDRs in the Icelandic study could be the result of the fact that Icelanders are more homogeneous genetically than most other European populations. In addition, differences in sample size may explain some of the discrepancies in shared familial risks, including the lack of an increased risk of ankylosing spondylitis among spouses. Extraintestinal manifestations have also been studied in a Swiss IBD cohort,^29^ including 580 patients with Crohn’s disease and 370 patients with ulcerative colitis, of whom 249 [43%] and 113 [31%] had at least one extraintestinal manifestation, including [6%] and [2%] with ankylosing spondylitis, respectively. Overall, extraintestinal manifestations, including ankylosing spondylitis, were more common in patients having a positive family history of IBD, but no control group was included for comparison.

### 4.3. Potential mechanisms and implications

Even though our findings of shared susceptibility between IBD and SpA do not reveal its nature, ie, genetic or environmental, similarities in magnitudes of associations within and across generations may point to shared genetics as the most prominent shared factor. Higher-magnitude associations in FDRs of patients with Crohn’s disease than with ulcerative colitis further support this hypothesis, since Crohn’s disease is known to have a larger genetic predisposition than ulcerative colitis.^9,30^ A role of shared genetic factors may also be reflected by the higher-magnitude associations found in FDRs of patients with early-onset IBD, which has a stronger genetic predisposition than late-onset IBD.^31^ Besides shared genetic risk factors, IBD and SpA may also have environmental risk factors in common. Active smoking is a risk factor for both Crohn’s disease and SpA,^32^ but has not been linked to an increased risk of ulcerative colitis. Smoking habits have also been reported to be familial.^33^ Thus, smoking may to some extent explain the observed increased risk of SpA in FDRs and spouses of patients with Crohn’s disease. Using a COPD diagnosis as crude proxy for smoking, we found that the shared familial risk between IBD and SpA was evident among spouses and FDRs without a COPD diagnosis only. These analyses were, however, limited in terms of not directly addressing the effect of smoking. Also, it is unlikely that smoking explains the entire risk, as an increased risk of SpA also was observed in FDRs and spouses of patients with ulcerative colitis.

From a clinical point of view, the observed differences in SpA rates in FDRs and spouses of patients with IBD, compared with FDRs and spouses of reference individuals, were rather small. Hence, our results do not to seem to support active screening of SpA among FDRs or spouses of IBD patients.

Although shared, early-life exposures, including delivery mode and infant feeding practices, significantly influence gut microbiota development among FDRs, common exposures among spouses, such as diet, probiotic use, and antibiotic use, may also alter its composition later in life. Fragoulis *et al*. reported that a dysregulated microbiome in the gut, and a migration of activated gut T cells and macrophages, may initiate inflammation in genetically predisposed individuals and activate inflammatory responses from the gut to the joint.^34^ An altered gut microbiota composition, including decreased bacterial diversity, has been observed in patients with IBD, especially Crohn’s disease, as well as those with SpA.^13,15,16,35,36^ The decrease in bacterial diversity is triggered by lower abundances of various taxa. The decline in relative abundance of Ruminococcus and Akkermansia was observed in patients with psoriatic arthritis.^15^ This is noteworthy, since the abundance of Ruminococcus is also decreased in IBD patients.^37^ Moreover, patients with Crohn’s disease and psoriatic arthritis have reduced abundances of another genus, Alistipes, commonly found in healthy populations.^15,37^ A recent study noted that patients with ankylosing spondylitis, and patients with IBD and ankylosing spondylitis, have gut microbiomes distinct from those of patients with IBD only and healthy individuals.^38^ However, they also reported enrichment of Streptococcus and Haemophilus in patients with either SpA or IBD and in patients having both diseases, suggesting the presence of shared microbiological triggers. Similarly, nutritional factors [including a poor vitamin D status] have been linked to both diseases.^18,19^ Evidence suggests that vitamin D may play a vital role in innate and acquired immunity, and its deficiency may make individuals more susceptible to severe disease in inflammatory disorders.^39^

Spouses are more likely to share environmental factors at an adult age, and exposure to such risk factors naturally increases with increasing age. Consequently, we may anticipate higher-magnitude associations in spouses with attained age, if associations in spouses are mainly driven by shared environmental factors. In this study we did not, however, find evidence of different-magnitude associations by attained age. Another potential explanation for the observed associations could be ascertainment bias, due to similarities in patterns of health-seeking behaviour. FDRs and spouses of IBD patients may seek medical advice more frequently compared with FDRs of reference individuals in the general population. Alternatively, physicians may be more likely to examine them for chronic inflammatory diseases.

### 4.4. Strengths and limitations

This study has several strengths. The population-based study design increases the generalisability of our findings, and the examination of a large, nationwide cohort allowed us to perform analyses across subsets of individuals. Data on family history and relationships were retrieved from nationwide registers with virtually complete follow-up. These strengths also minimise information and selection biases. To accurately define the date of first diagnosis of IBD we used information from the ESPRESSO pathology database. Compared with the Icelandic study,^11^ this study was performed in a population that is more representative of Europeans in general, and as such provides risk estimates that are likely to be generalisable to a larger population.

Our study also has potential limitations. Despite large numbers of FDRs and spouses of patients with IBD, we still had limited precision and power in some subgroup analyses, such as in FDRs of patients aged <18 years at the diagnosis of IBD, and when limiting follow-up to time since diagnosis of IBD. Due to the nationwide, register-based design, we did not have information on possible risk factors, such as smoking and dietary factors, which could potentially explain the different patterns of associations observed in FDRs and spouses. We could only indirectly address the potential role of smoking using COPD diagnosis as a crude proxy, since Swedish national health care registers lack smoking data. Likewise, we could not explore the potential role of health-seeking behaviour in the observed associations, as we lacked access to primary care data.

Moreover, we were unable to confirm diagnoses of IBD and SpA through medical record review, and misclassifications of the diagnoses cannot be excluded. However, previous studies showed that the positive predictive value [PPV] for IBD with two or more relevant ICD codes in the NPR was 93%,^40^ and 95% with one patient register record documenting IBD and a colorectal biopsy showing inflammation.^41,42^ Based on ≥1 ICD code, the PPV for ankylosing spondylitis has been reported to be 70% when applying the modified New York criteria, 89% for any set of SpA criteria,^24^ and 63–92% for psoriatic arthritis.^25,26^ In addition, we included spouses based on a record of marriage alone, as no other data on cohabitation or divorce were available in the used registries. Since we could not censor spouses who separated later in life, this could potentially have biased associations towards the null, particularly in a country like Sweden, where cohabitation without marriage is common and the divorce rate is high [2.3 divorces per 1000 persons in 2021].^43^ Moreover, since our analysis did not include individuals cohabiting without being married, which is increasingly common these days, results of this analysis are not necessarily generalisable to cohabiting individuals in general. Last, although our sensitivity analyses demonstrated robust results, we cannot completely rule out residual bias.

### 4.5. Conclusions

In conclusion, we found evidence of shared familial risks between IBD and SpA, as reflected by an increased risk of SpA in FDRs of IBD patients as compared with FDRs of matched reference individuals from the general population, and with this relative risk being most pronounced among FDRs of patients with Crohn’s disease and those with IBD diagnosed at an early age. These findings support a common immune pathogenesis of both diseases and demonstrate the importance of shared genetic factors. However, shared exposure to environmental risk factors also seems to play a role, since spouses of patients with IBD were also at increased risk for SpA. Another potential explanation for the increased risk in spouses could be ascertainment bias because of similarities in patterns of health-seeking behaviour.

## Supplementary Data

Supplementary data are available at *ECCO-JCC* online.

jjae041_suppl_Supplementary_Figures_S1-S2_Tables_S1-S9

## Data Availability

The data underlying this article cannot be shared publicly due to privacy of individuals who participated in the study.
