# Supplementary material for: Spondyloarthritis in First-Degree Relatives and Spouses of Patients with Inflammatory Bowel Disease: A Nationwide Population-based Cohort Study from Sweden
Source: J Crohns Colitis. 2024 Mar 22;18(9):1371–80. doi: 10.1093/ecco-jcc/jjae041 (PMC11369065; doi:10.1093/ecco-jcc/jjae041)
Supplement: jjae041_suppl_Supplementary_Figures_S1-S2_Tables_S1-S9 [file jjae041_suppl_supplementary_figures_s1-s2_tables_s1-s9.docx]

**Spondyloarthritis in first-degree relatives and spouses of patients with inflammatory bowel disease: A nationwide population-based cohort study from Sweden**

Page 1. **Content**

Page 2. **Supplementary Table 1:** International Classification of Diseases [ICD] codes for inflammatory bowel

Disease.

Page 3. **Supplementary Table 2.** International Classification of Diseases [ICD] codes for spondyloarthritis.

Page 4. **Supplementary Table 3.** Risk of SpA comparing siblings / spouses of IBD patients to siblings / spouses of reference individuals from the general population, overall and by subtype: with follow-up from 1 January 2001.

Page 5. **Supplementary Table 4**. Risk of SpA comparing siblings / spouses of IBD patients to siblings / spouses of reference individuals from the general population, stratified by age and sex of IBD index case / reference individuals: with follow-up from 1 January 2001.

Page 6. **Supplementary Table 5.** Risk of SpA from the index date comparing siblings / spouses of IBD patients to siblings / spouses of reference individuals from the general population, stratified by sex and age of IBD index patient / reference individual.

Page 7. **Supplementary Table 6.** Risk estimates of SpA comparing IBD-free FDRs and spouses of IBD patients to IBD-free FDRs and spouses of reference individuals.

Page 8. **Supplementary Table 7.** Risk estimates of SpA comparing FDRs / spouses vs FDRs / spouses of reference individuals of Crohn’s disease patients and ulcerative colitis patients with/without COPD diagnoses.

Page 9. **Supplementary Table 8.** Risk estimates of ankylosing spondylitis comparing FDRs and spouses of IBD patients to FDRs and spouses of reference individuals, overall and by subtype.

Page 10. **Supplementary Table 9.** Risk estimates of ankylosing spondylitis comparing FDRs and spouses of IBD patients to FDRs and spouses of reference individuals, stratified by age and sex of IBD index case / reference individuals.

Page 11. **Supplementary Figure 1.** Flow chart for selection of study population.

Page 12. **Supplementary Figure 2.** Study design.

Page 13. **Supplementary Methods**

Page 14. **References**

**Supplementary Table 1.** International Classification of Disease [ICD] codes and SNOMED codes defining inflammatory bowel diseases [IBD].^1^

|  | ICD 10  1997- | ICD 9  1987-1996 | SNOMED codes |
| --- | --- | --- | --- |
| Crohn's disease | K50 | 555 | D6216 or M41, M42, M43, M44, M463, or M47 |
| Ulcerative colitis | K51 | 556 | D6255 or M41, M42, M43, M44, or M47 |
| Inflammatory bowel disease unclassified | UC + CD or K52.3 | UC+CD | D6214 or M41, M42, M43, M44, M463, or M47 |

1. Everhov AH, Halfvarson J, Myrelid P, Sachs MC, Nordenvall C, Soderling J, et al. Incidence and Treatment of Patients Diagnosed With Inflammatory Bowel Diseases at 60 Years or Older in Sweden. Gastroenterology. 2018;154(3):518-28 e15.

Abbreviations: ICD = international classification of diseases; SNOMED = systemized nomenclature of medicine; CD = Crohn's disease; UC = ulcerative colitis

**Supplementary Table 2:** International Classification of Disease [ICD] codes for spondyloarthropathies [SpA].

|  | ICD 10  1997- | ICD 9  1987-1996 |
| --- | --- | --- |
| Spondyloarthritis | M45, M08.1  L40.5, M07.0-3, M07.4-5, M07.6, M09.1.2  M02.0-2, M02.3, M02.8-9  M46.0/M46.1/8/9 | 720A  696A, 713B  099.D, 711A  720B, 720C, 720W |
| Ankylosing spondylitis | M45, M08.1 | 720A |
| Psoriatic and enteropathic arthritis | L40.5, M07.0-3, M07.4-5, M07.6, M09.1.2 | 696A, 713B |
| Reactive arthritis | M02.0-2, M02.3, M02.8-9 | 099.D, 711A |
| Undifferentiated SpA/ Sacroiliitis | M46.0/M46.1/8/9 | 720B, 720C, 720W |

**Supplementary Table 3.** Risk of SpA comparing siblings / spouses of IBD patients to siblings / spouses of reference individuals from the general population, overall and by subtype: with follow-up from 1 January 2001.

| Exposure | Siblings of  IBD patients  (N with SpA / N total) | Siblings of  Reference individuals  (N with SpA / N total) | HR [95% CI] | Spouses of  IBD patients  (N with SpA / N total) | Spouses of  Reference individuals  (N with SpA / N total) | HR [95% CI] |
| --- | --- | --- | --- | --- | --- | --- |
| IBD | 758 / 48,528 | 5,449 / 476,936 | 1.37 [1.27, 1.48] | 284 / 25,247 | 2,388 / 252,599 | 1.20 [1.06, 1.35] |
| Crohn's disease | 247 / 14,256 | 1,553 / 141,882 | 1.60 [1.40, 1.83] | 76 / 6,943 | 637 / 69,272 | 1.20 [0.94, 1.52] |
| Ulcerative colitis | 378 / 25,121 | 2,814 / 246,215 | 1.31 [1.18, 1.46] | 148 / 13,634 | 1,290 / 136,647 | 1.16 [0.98, 1.37] |

Hazard ratios were obtained from Cox proportional hazards models with follow-up from 1 January 2001 and attained age as underlying time scale, adjusting for sex and 10-year calendar period at birth date.

Abbreviations: SpA = spondyloarthritis; FDRs = first-degree relatives; HR = hazard ratio; CI = confidence interval; N = number; IBD = inflammatory bowel disease.

**Supplementary Table 4.** Risk of SpA comparing siblings / spouses of IBD patients to siblings / spouses of reference individuals from the general population, stratified by age and sex of IBD index case / reference individuals, with follow-up from 1 January 2001.

| **Exposure** |  | **Siblings of**  **IBD patients**  (N with SpA / N total) | **Siblings of**  **Reference individuals**  (N with SpA / N total) | **HR [95% CI]** | **Spouses of**  **IBD patients**  (N with SpA / N total) | **Spouses of**  **Reference individuals**  (N with SpA / N total) | **HR [95% CI]** |  |
| --- | --- | --- | --- | --- | --- | --- | --- | --- |
| **IBD** | **Age group** |  |  |  |  |  |  |  |
|  | <18 years | 31 / 5,566 | 194 / 57,086 | 1.58 [1.08, 2.32] | NA | NA | NA |  |
|  | 18-60 years | 539 / 33,243 | 3,755 / 325,668 | 1.41 [1.29, 1.55] | 202 / 18,158 | 1,656 / 182,546 | 1.24 [1.07, 1.43] |  |
|  | >60 years | 188 / 9,719 | 1,500 / 94,182 | 1.23 [1.05, 1.43] | 82 / 6,966 | 728 / 68,807 | 1.11 [0.88, 1.40] |  |
|  | P interaction |  |  | 0.21 |  |  | 0.22 |  |
|  | **Sex** |  |  |  |  |  |  |  |
|  | Female | 392 / 23,814 | 2,752 / 231,539 | 1.38 [1.24, 1.53] | 140 / 12,974 | 1,139 / 128,670 | 1.23 [1.03, 1.46] |  |
|  | Male | 366 / 24,714 | 2,697 / 245,397 | 1.36 [1.22, 1.51] | 144 / 12,273 | 1,249 / 123,929 | 1.17 [0.98, 1.39] |  |
|  | P interaction |  |  | 0.87 |  |  | 0.69 |  |
| **Crohn's disease** | **Age group** |  |  |  |  |  |  |  |
|  | <18 years | 11 / 2,285 | 78 / 2,3012 | 1.42 [0.75, 2.66] | NA | NA | NA |  |
|  | 18-60 years | 181 / 9,470 | 1,049 / 93,869 | 1.74 [1.49, 2.04] | 54 / 5,042 | 443 / 50,896 | 1.25 [0.94, 1.65] |  |
|  | >60 years | 55 / 2,501 | 426 / 25,001 | 1.30 [0.98, 1.72] | 22 / 1,851 | 192 / 17,854 | 1.10 [0.71, 1.72] |  |
|  | P interaction |  |  | 0.17 |  |  | 0.42 |  |
|  | **Sex** |  |  |  |  |  |  |  |
|  | Female | 135 / 7,198 | 823 / 71,328 | 1.63 [1.36, 1.95] | 38 / 3,796 | 316 / 37,204 | 1.17 [0.84, 1.64] |  |
|  | Male | 112 / 7,058 | 730 / 70,554 | 1.57 [1.28, 1.91] | 38 / 3,147 | 321 / 32,068 | 1.21 [0.87, 1.70] |  |
|  | P interaction |  |  | 0.81 |  |  | 0.92 |  |
| **Ulcerative colitis** | **Age group** |  |  |  |  |  |  |  |
|  | <18 years | 13 / 2,100 | 82 / 21,825 | 1.57 [0.88, 2.82] | NA | NA | NA |  |
|  | 18-60 years | 266 / 17,902 | 1,991 / 175,215 | 1.31 [1.16, 1.49] | 105 / 9,976 | 912 / 99,967 | 1.16 [0.95, 1.42] |  |
|  | >60 years | 99 / 5,119 | 741 / 49,175 | 1.29 [1.04, 1.59] | 43 / 3,612 | 376 / 36,185 | 1.14 [0.83, 1.57] |  |
|  | P interaction |  |  | 0.82 |  |  | 0.51 |  |
|  | **Sex** |  |  |  |  |  |  |  |
|  | Female | 193 / 25,121 | 1421 / 118,382 | 1.31 [1.13, 1.52] | 72 / 6,899 | 590 / 68,536 | 1.23 [0.96, 1.57] |  |
|  | Male | 185 / 12,925 | 1393 / 127,833 | 1.32 [1.13, 1.54] | 76 / 6,735 | 700 / 68,111 | 1.10 [0.87, 1.39] |  |
|  | P interaction |  |  | 0.95 |  |  | 0.53 |  |

Hazard ratios were obtained from Cox proportional hazards models with follow-up from 1 January 2001 with attained age as underlying time scale, adjusting for sex and 10-year calendar period at birth date.

Abbreviations: SpA = spondyloarthritis; HR = hazard ratio; CI = confidence interval; N = number; IBD = inflammatory bowel disease.

**Supplementary Table 5.** Risk of SpA from the index date comparing siblings / spouses of IBD patients to siblings / spouses of reference individuals from the general population, stratified by sex and age of IBD index patient / reference individual.

| **Exposure** |  | **Siblings of**  **IBD patients**  (N with SpA / N total) | **Siblings of**  **Reference individuals**  (N with SpA / N total) | **HR [95% CI]** |  | **Spouses of**  **IBD patients**  (N with SpA / N total) | **Spouses of**  **Reference individuals**  (N with SpA / N total) | **HR [95% CI]** |
| --- | --- | --- | --- | --- | --- | --- | --- | --- |
| **IBD** | **Age group** |  |  |  |  |  |  |  |
|  | <18 years | 16 / 5,367 | 108 / 54,994 | 1.49 [0.88, 2.52] |  | NA | NA | NA |
|  | 18-60 years | 206 / 31,674 | 1,392 / 311,191 | 1.46 [1.26, 1.69] |  | 77 / 17,552 | 631 / 177,397 | 1.24 [0.98, 1.57] |
|  | >60 years | 46 / 8,622 | 392 / 84,638 | 1.15 [0.85, 1.57] |  | 21 / 6,522 | 194 / 64,301 | 1.07 [0.68, 1.67] |
|  | P interaction |  |  | 0.36 |  |  |  | 0.38 |
|  | **Sex** |  |  |  |  |  |  |  |
|  | Female | 135 / 22,409 | 935 / 218,639 | 1.41 [1.18, 1.69] |  | 47 / 12,340 | 374 / 122,914 | 1.25 [0.92, 1.69] |
|  | Male | 133 / 23,254 | 957 / 232,184 | 1.39 [1.16, 1.67] |  | 51 / 11,855 | 453 / 120,022 | 1.15 [0.86, 1.53] |
|  | P interaction |  |  | 0.89 |  |  |  | 0.68 |
| **Crohn's disease** | **Age group** |  |  |  |  |  |  |  |
|  | <18 years | 6 / 2,185 | 45 / 22,213 | 1.36 [0.58, 3.19] |  | NA | NA | NA |
|  | 18-60 years | 69 / 9,019 | 382 / 89,769 | 1.83 [1.41, 2.36] |  | 19 / 4,870 | 159 / 49,403 | 1.22 [0.76, 1.96] |
|  | >60 years | 11 / 2,229 | 123 / 22,433 | 0.89 [0.48, 1.66] |  | 7 / 1,728 | 46 / 16,630 | 1.45 [0.65, 3.20] |
|  | P interaction |  |  | 0.08 |  |  |  | 0.55 |
|  | **Sex** |  |  |  |  |  |  |  |
|  | Female | 46 / 6,778 | 279 / 67,460 | 1.64 [1.20, 2.24] |  | 13 / 3,618 | 99 / 35,534 | 1.27 [0.71, 2.26] |
|  | Male | 40 / 6,655 | 271 / 66,955 | 1.51 [1.08, 2.10] |  | 13 / 3,029 | 107 / 31,018 | 1.25 [0.71, 2.23] |
|  | P interaction |  |  | 0.68 |  |  |  | 0.96 |
| **Ulcerative colitis** | **Age group** |  |  |  |  |  |  |  |
|  | <18 years | 8 / 2,040 | 41 / 21,023 | 1.98 [0.93, 4.22] |  | NA | NA | NA |
|  | 18-60 years | 104 / 17,033 | 755 / 167,362 | 1.36 [1.10, 1.66] |  | 47 / 9,647 | 360 / 97,251 | 1.32 [0.97, 1.79] |
|  | >60 years | 28 / 4,557 | 181 / 44,191 | 1.50 [1.01, 2.23] |  | 11 / 3,400 | 102 / 33,901 | 1.08 [0.58, 2.00] |
|  | P interaction |  |  | 0.59 |  |  |  | 0.45 |
|  | **Sex** |  |  |  |  |  |  |  |
|  | Female | 68 / 23,630 | 486 / 111,754 | 1.37 [1.06, 1.76] |  | 28 / 6,566 | 205 / 65,636 | 1.37 [0.92, 2.03] |
|  | Male | 72 / 12,148 | 491 / 120,822 | 1.45 [1.13, 1.86] |  | 30 / 6,526 | 258 / 66,008 | 1.17 [0.80, 1.71] |
|  | P interaction |  |  | 0.71 |  |  |  | 0.59 |
| Hazard ratios of SpA comparing siblings / spouses of IBD patients to siblings / spouses of reference individuals were obtained from Cox proportional hazards models with time from the index date as underlying time scale, adjusting for age, sex, and calendar year at index date (years). Abbreviations: SpA = spondyloarthritis; IBD = inflammatory bowel disease; HR = hazard ratio; CI = confidence interval; N = number; IBD = inflammatory bowel disease. | | | | | | | | |

**Supplementary Table 6.** Risk estimates of SpA comparing IBD-free FDRs and spouses of IBD patients to IBD-free FDRs and spouses of reference individuals.

| **Exposure** | **IBD-free FDRs of IBD patients**  N with SpA / N total | **IBD-free FDRs of Reference individuals**  N with SpA / N total | **HR [95% CI]** | **IBD-free Spouses of IBD patients**  N with SpA / N total | | **IBD-free Spouses of Reference individuals**  N with SpA / N total | | **HR [95% CI]** | |
| --- | --- | --- | --- | --- | --- | --- | --- | --- | --- |
| **IBD** | 2,020 / 141,358 | 16,477 / 1,433,759 | 1.24 [1.19, 1.30 ] | | 288 / 25,480 | | 2,399 / 254,039 | | 1.21 [1.07, 1.37] |
| **Crohn's disease** | 589 / 40,591 | 4,727 / 418,451 | 1.29 [1.18, 1.40 ] | | 74 / 6,985 | | 645 /69,707 | | 1.15 [0.91, 1.47] |
| **Ulcerative colitis** | 1,047 / 74,207 | 8,563 / 750,631 | 1.24 [1.16, 1.32 ] | | 153/13,762 | | 1,289 / 137,429 | | 1.20 [1.01, 1.42] |

Hazard ratios were obtained from Cox proportional hazards models with attained age as underlying time scale, adjusting for sex, and 10-year calendar period at birth date.

Abbreviations: SpA = spondyloarthritis; FDR = first-degree relative, HR = hazard ratio; CI = confidence interval; N = number; IBD = inflammatory bowel disease.

**Supplementary Table 7.** Risk estimates of SpA comparing spouses of patients with inflammatory bowel disease, Crohn's disease and ulcerative colitis to spouses of reference individuals from the general population without a COPD diagnosis.

| **Exposure** | **FDRs of**  **IBD patients**  (N with SpA / N total) | **FDRs of**  **Reference individuals**  (N with SpA / N total) | **HR [95% CI]** |  | **Spouses of**  **IBD patients**  (N with SpA / N total) | **Spouses of**  **Reference individuals**  (N with SpA / N total) | **HR [95% CI]** |
| --- | --- | --- | --- | --- | --- | --- | --- |
| **IBD without COPD** | 2,303 / 141,457 | 17,274 / 1,425,406 | 1.35 [1.29, 1.40] |  | 309 / 24,591 | 2,600 / 251,520 | 1.23 [1.10, 1.39] |
| **Crohn's disease without COPD** | 710 / 40,768 | 4,986 /416,856 | 1.46 [1.35, 1.58] |  | 79/ 6,734 | 706 /69,145 | 1.16 [0.92, 1.47] |
| **Ulcerative colitis without COPD** | 1,178 / 74,520 | 8,974 / 745,913 | 1.31 [1.23, 1.39] |  | 163 / 13,385 | 1400 / 136,104 | 1.20 [1.02, 1.42] |
| Hazard ratios were obtained from Cox proportional hazards models with attained age as underlying time scale, adjusting for sex and 10-year calendar period at birth date.  Abbreviations: SpA = spondyloarthritis; CI = confidence interval; FDRs = first-degree relatives; HR = hazard ratio; N = number; IBD = Inflammatory bowel disease | | | | | | | |

**Supplementary Table 8.** Risk estimates of ankylosing spondylitis comparing FDRs and spouses of IBD patients to FDRs and spouses of reference individuals, overall and by subtype.

| **Exposure** | **FDRs of**  **IBD patients**  N with AS / N total | **FDRs of**  **Reference individuals**  N with AS / N total | **HR [95% CI]** | **Spouses of**  **IBD patients**  N with AS / N total | **Spouses of**  **Reference individuals**  N with AS / N total | **HR [95% CI]** |
| --- | --- | --- | --- | --- | --- | --- |
| **IBD** | 617 / 147,047 | 3,878 / 1,452,882 | 1.57 [1.44, 1.71] | 85 / 25,945 | 626 / 258,098 | 1.36 [1.08, 1.70] |
| **Crohn's disease** | 216 / 42,354 | 1,074 / 423,863 | 2.01 [1.74, 2.33] | 18 / 7,125 | 183 / 70,832 | 0.98 [0.60, 1.59] |
| **Ulcerative colitis** | 302 / 77,194 | 2,029 / 760,723 | 1.46 [1.29, 1.65] | 47 / 14,019 | 324 / 139,592 | 1.45 [1.07, 1.97] |

Hazard ratios were obtained from Cox proportional hazards models with attained age as underlying time scale, adjusting for sex, and 10-year calendar period at birth date.

Abbreviations: SpA = spondyloarthritis; FDR = first-degree relative, HR = hazard ratio; CI = confidence interval; N = number; IBD = inflammatory bowel disease; AS = ankylosing spondylitis.

**Supplementary Table 9.** Risk estimates of ankylosing spondylitis comparing FDRs and spouses of IBD patients to FDRs and spouses of reference individuals, stratified by age and sex of IBD index case / reference individuals.

| **Exposure** |  | **FDRs of**  **IBD patients**  N with AS / N total | **FDRs of**  **Reference individuals**  N with AS / N total | **HR [95% CI]** | **Spouses of**  **IBD patients**  N with AS / N total | | **Spouses of**  **Reference individuals**  N with AS / N total | | **HR [95% CI]** |  | |
| --- | --- | --- | --- | --- | --- | --- | --- | --- | --- | --- | --- |
| **IBD** | **Age group** |  |  |  | |  |  |  | | |  |
|  | <18 years | 51 / 13,936 | 320 / 139,404 | 1.56 [1.16, 2.10] | | NA | NA | NA | | |  |
|  | 18-60 years | 437 / 105,821 | 2,624 / 1,044,751 | 1.64 [1.48, 1.82] | | 54 / 18,622 | 430 / 185,857 | 1.27 [0.95, 1.68] | | |  |
|  | >60 years | 129 / 27,290 | 934 / 268,727 | 1.35 [1.13, 1.63] | | 31 / 7200 | 195 / 70,980 | 1.57 [1.07, 2.29] | | |  |
|  | P interaction |  |  | 0.17 | |  |  | 0.32 | | |  |
|  | **Sex** |  |  |  | |  |  |  | | |  |
|  | Female | 322 / 74,246 | 1954 / 729,356 | 1.60 [1.42, 1.80] | | 53 / 13,375 | 370 / 131,910 | 1.42 [1.07, 1.90] | | |  |
|  | Male | 295 / 72,801 | 1924 / 723,526 | 1.53 [1.35, 1.73] | | 32 / 12,570 | 256 / 126,188 | 1.27 [0.88, 1.83] | | |  |
|  | P interaction |  |  | 0.60 | |  |  | 0.61 | | |  |
| **Crohn's disease** | **Age group** |  |  |  | |  |  |  | | |  |
|  | <18 years | 19 / 5,642 | 123 / 56,251 | 1.53 [0.94, 2.48] | | NA | NA | NA | | |  |
|  | 18-60 years | 149 / 29,701 | 692 / 298,289 | 2.16 [1.81, 2.58] | | 10 / 5,158 | 126 / 51,864 | 0.80 [0.42, 1.53] | | |  |
|  | >60 years | 48 / 7,011 | 259 / 69,323 | 1.84 [1.35, 2.50] | | 8 / 1,917 | 57 / 18,440 | 1.36 [0.65, 2.84] | | |  |
|  | P interaction |  |  | 0.31 | |  |  | 0.30 | | |  |
|  | **Sex** |  |  |  | |  |  |  | | |  |
|  | Female | 124 / 22,060 | 533 / 219,480 | 2.29 [1.88, 2.78] | | 10 / 3,906 | 107 / 38,198 | 0.91 [0.48, 1.74] | | |  |
|  | Male | 92 / 20,294 | 541 / 204,383 | 1.73 [1.39, 2.16] | | 8 / 3,219 | 76 / 32,634 | 1.08 [0.52, 2.23] | | |  |
|  | P interaction |  |  | 0.07 | |  |  | 0.72 | | |  |
| **Ulcerative colitis** | **Age group** |  |  |  | |  |  |  | | |  |
|  | <18 years | 25 / 5,311 | 132 / 53,394 | 1.86 [1.21, 2.85] | | NA | NA | NA | | |  |
|  | 18-60 years | 223 / 57,515 | 1,425 / 565,840 | 1.54 [1.34, 1.77] | | 30 / 10,243 | 229 / 101,763 | 1.31 [0.90, 1.92] | | |  |
|  | >60 years | 54 / 14,368 | 472 / 141,489 | 1.11 [0.84, 1.48] | | 17 / 3,730 | 94 / 37,327 | 1.79 [1.07, 3.01] | | |  |
|  | P interaction |  |  | 0.07 | |  |  | 0.28 | | |  |
|  | **Sex** |  |  |  | |  |  |  | | |  |
|  | Female | 153 / 38,603 | 1,063 / 378,985 | 1.40 [1.18, 1.66] | | 33 / 7,118 | 191 / 70,201 | 1.73 [1.19, 2.50] | | |  |
|  | Male | 149 / 38,591 | 966 / 381,738 | 1.53 [1.29, 1.82] | | 14 / 6,901 | 133 / 69,391 | 1.07 [0.61, 1.85] | | |  |
|  | P interaction |  |  | 0.48 | |  |  | 0.14 | | |  |

Hazard ratios of SpA comparing FDRs / spouses of IBD cases to FDRs / spouses of reference individuals were obtained from Cox proportional hazards models with attained age as underlying time scale, adjusting for sex, and 10-year calendar period at birth date.

Abbreviations: SpA = spondyloarthritis; FDR = first-degree relative; HR = hazard ratio; CI = confidence interval; N = number; NA = not available; AS = ankylosing spondylitis; IBD = inflammatory bowel disease.

**Supplementary Figure 1**. Flow chart for selection of study population.

Records identified through the NPR, ESPRESSO, and the Swedish Total Population Register:

Index IBD patients [n = 39,203],

Reference individuals [n=390,490]

Identification

FDRs who were born before 1932

Excluded:

FDRs [n=209,296]

Spouses [n =17,956]

Individuals with missing/incorrect information on emigration or death date before 1987

Excluded:

FDRs who emigrated [n=18,184], FDRs who died [n=14,947]

Spouses who emigrated [n=4,349], spouses who died [n=593]

Individuals with missing/incorrect information on age of FDRs/spouses

Excluded:

FDRs [n=1,553], Spouses [n=74]

Eligibility

Included

**Supplementary Figure 1**. Flow chart for selection of study population in Sweden. Abbreviations: ESPRESSO = epidemiology strengthened by histopathology reports in Sweden; IBD = inflammatory bowel disease; ICD = international classification of diseases; N = number; NPR = national patient register, FDRs = first-degree relatives

Records after exclusion of missing/incorrect information:

FDRs of IBD patients [n =147,080], FDRs of Reference individuals [n=1,453,429]

Spouses of IBD patients [n=25,945], Spouses of Reference individuals [n=258,098]

Records after exclusion of missing/incorrect information:

FDRs of IBD patients [n=147,242], FDRs of reference individuals [n=1,454,820]

Spouses of IBD patients [n=25,953], Spouses of reference individuals [n=258,164]

Records after exclusion of FDRs born before 1932:

FDRs of IBD patients [n=150,901], FDRs of reference individuals [n=1,484,292]

Spouses of IBD patients [n=26,522], Spouses of reference individuals [n=262,537]

After linkage with multi-generation register and total population register:

FDRs of IBD patients [n=169,782], FDRs of reference individuals [n=1,674,707]

Spouses of IBD patients [n=28,210], spouses of reference individuals [n=278,805]

**Supplementary Figure 2:** Study design: Cohort study, we estimated rates incident diagnosis of Spondyloarthritis (SpA) in first-degree relatives (FDRs) and spouses of patients diagnosed with IBD 2006-2016 and of matched reference individuals from the general population. We used Cox proportional hazard regression models to estimate hazard ratios (HRs).


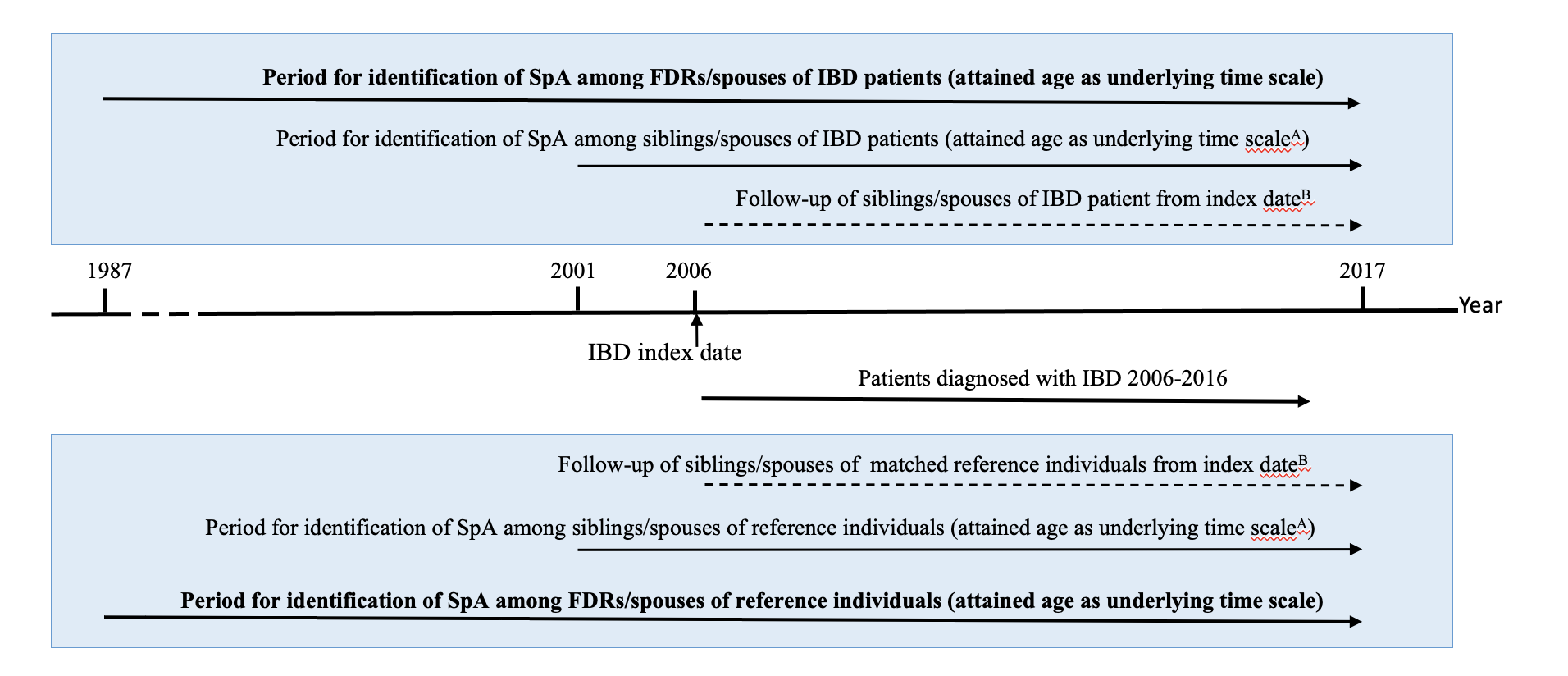


^A^ Since data on non-primary outpatient care were missing in the National Patient Registry before 2001, we repeated analyses using 1 January 1, 2001, as the start of follow-up. These latter analyses were performed to reflect modern diagnostic criteria and minimise information bias.

^B^ To facilitate the interpretation of risks from a clinical perspective and avoid misclassification due to calendar period effects across generations, include siblings and spouses of IBD patients/reference individuals only, and shift the start of follow-up to the index date.

**Supplementary methods**

Swedish Multi-generation Register was produced by Statistics Sweden using the Swedish Total Population Register [TPR] that was initiated in 1968. The Swedish Multi-generation Register includes information on people born from 1932 onwards and any relationship to biological and adoptive parents and those who are registered as residents in Sweden after 1961 (1). Parents, children, and their siblings can be linked through the Register data.

**References**

1. Ekbom A. The Swedish Multi-generation Register. Methods Mol Biol. 2011;675:215, 20.

Risk of SpA from the index date comparing siblings / spouses of IBD patients to siblings / spouses of reference individuals from the general population, stratified by sex and age of IBD index patient / reference individual.
